# Structure-Function Analysis of the GlyR α2 Subunit Autism Mutation p.R323L Reveals a Gain-of-Function

**DOI:** 10.3389/fnmol.2017.00158

**Published:** 2017-05-23

**Authors:** Yan Zhang, Thi Nhu Thao Ho, Robert J. Harvey, Joseph W. Lynch, Angelo Keramidas

**Affiliations:** ^1^Queensland Brain Institute, The University of QueenslandBrisbane, QLD, Australia; ^2^Department of Pharmacology, UCL School of PharmacyLondon, United Kingdom; ^3^School of Biomedical Sciences, The University of QueenslandBrisbane, QLD, Australia

**Keywords:** autism spectrum disorder, epilepsy, glycine receptor, *GLRA2*, GlyR α2 subunit

## Abstract

Glycine receptors (GlyRs) containing the α2 subunit regulate cortical interneuron migration. Disruption of the GlyR α2 subunit gene (*Glra2*) in mice leads to disrupted dorsal cortical progenitor homeostasis, leading to a depletion of projection neurons and moderate microcephaly in newborn mice. In humans, rare variants in *GLRA2*, which is located on the X chromosome, are associated with autism spectrum disorder (ASD) in the hemizygous state in males. These include a microdeletion (*GLRA2*∆ex8-9) and missense mutations in *GLRA2* (p.N109S and p.R126Q) that impair cell-surface expression of GlyR α2, and either abolish or markedly reduce sensitivity to glycine. We report the functional characterization of a third missense variant in *GLRA2* (p.R323L), associated with autism, macrocephaly, epilepsy and hypothyroidism in a female proband. Using heterosynapse and macroscopic current recording techniques, we reveal that GlyR α2^R323L^ exhibits reduced glycine sensitivity, but significantly increased inhibitory postsynaptic current (IPSC) rise and decay times. Site-directed mutagenesis revealed that the nature of the amino acid switch at position 323 is critical for impairment of GlyR function. Single-channel recordings revealed that the conductance of α2^R323L^β channels was higher than α2β channels. Longer mean opening durations induced by p.R323L may be due to a change in the gating pathway that enhances the stability of the GlyR open state. The slower synaptic decay times, longer duration active periods and increase in conductance demonstrates that the GlyR α2 p.R323L mutation results in an overall gain of function, and that GlyR α2 mutations can be pathogenic in the heterozygous state in females.

## Introduction

Glycine receptors (GlyRs) are key members of a ligand-gated ion channel superfamily that includes nicotinic acetylcholine receptors (nAChRs), 5-hydroxytryptamine type-3 receptors (5-HT_3_Rs) and γ-aminobutyric acid type-A receptors (GABA_A_Rs). There are four GlyR α subunits (α1–α4) and one β subunit, that share a common topology: a large N-terminal extracellular domain (ECD) that harbors the ligand-binding site and four transmembrane domains (M1–M4) connected by short intracellular (M1–M2) and extracellular (M2–M3) loops and a long intracellular loop connecting M3 to M4 (Lynch, 2009). GlyRs can be formed as homomers, consisting of α subunits only, or as heteromers comprising α and β subunits in a 3α:2β or 2α:3β stoichiometry (Grudzinska et al., 2005; Durisic et al., 2012; Yang et al., 2012). Key biological roles of different GlyR isoforms have been revealed by the study of GlyR dysfunction in rodent models and human disease. For example, the major adult GlyR isoform consisting of α1 and β GlyR subunits, has a major role in the control of spinal motor reflex circuits. Mutations in the genes encoding this GlyR subtype (*GLRA1* and *GLRB*) cause startle disease, characterized by noise- or touch-induced non-epileptic seizures, excessive muscle stiffness and neonatal apnea episodes in cattle, mice and humans (Harvey et al., 2008; Bode and Lynch, 2014). Allelic variants of *GLRB* have also recently been associated with agoraphobic behavior, an increased startle response and fear network activation (Deckert et al., 2017).

By contrast, GlyR α3 subunit knockout mice have revealed a role for this subtype in central inflammatory pain sensitization (Harvey et al., 2004), rhythmic breathing (Manzke et al., 2010), ethanol intake, preference and taste aversion (Blednov et al., 2015) and auditory nerve function (Dlugaiczyk et al., 2016). The GlyR α4 subunit has been linked to neurotransmitter release in sympathetic neurons (Boehm et al., 1997; Harvey et al., 2000) but is thought to be a pseudogene in humans (Simon et al., 2004) due to a stop codon in *GLRA4* exon 9, causing a protein truncation between membrane-spanning domains M3 and M4. Perhaps for this reason, no mouse knockout model currently exists.

The GlyR α2 subtype has previously been linked to roles in synaptogenesis (Kirsch and Betz, 1998; Levi et al., 1998), cell fate and paracrine transmitter release (Mangin et al., 2003) in the developing cortex and spinal cord (Flint et al., 1998; Scain et al., 2010). GlyR α2 is also pivotal in the modulation of ethanol intake, aversion and preference (Blednov et al., 2015), retinal photoreceptor development (Young and Cepko, 2004) and the control of receptive field surround in retinal ganglion cells (Nobles et al., 2012; Zhang C. et al., 2015). However, more recent studies using a novel *Glra2* knockout line provided compelling evidence that extrasynaptic activation of GlyRs containing the α2 subunit in interneurons is vital for control of cortical tangential migration during embryogenesis (Avila et al., 2013). In *Glra2* knockout mice, dorsal cortical progenitor homeostasis was disrupted (Avila et al., 2014) impairing the capacity of apical progenitors to generate basal progenitors. This resulted in a reduction of projection neurons in upper or deep layers of the cerebral cortex and moderate microcephaly in newborn *Glra2* knockout mice (Avila et al., 2014). Somatosensory cortical neurons in *Glra2* knockout mice also have more dendritic branches with an overall increase in total spine number. This results in disruption of the excitation/inhibition balance, with an overall increase network excitability and enhanced susceptibility to epileptic seizures (Morelli et al., 2017) as well as defects in long-term potentiation and object recognition memory (Pilorge et al., 2016).

The kinetic properties of homomeric α2 subunit GlyRs have been studied at the single-channel and macropatch levels (Mangin et al., 2003; Krashia et al., 2011). These studies reveal that wild-type homomeric α2 GlyRs activate for longer durations than α1-containing GlyRs (Krashia et al., 2011) and activate and deactivate more slowly on an ensemble macropatch level (Mangin et al., 2003). Changes in GlyR subunit mRNA levels suggest a developmental switch in expression from predominantly α2 in embryonic/neonatal rodents to α1/α3 in juveniles/adults, whereas expression of the β subunit remains high throughout this period of development. Coupled with the observation that synaptic current decay is relatively slow in neonatal neurons and accelerates in neurons of juvenile rodents, it is reasonable to infer a developmental switch from α2 homomers or α2β heteromers to heteromeric α1β or α3β GlyRs (Singer et al., 1998).

Consistent with these findings, microdeletions and missense mutations in the human GlyR α2 subunit gene (*GLRA2*), which is located on the X-chromosome, have been associated with rare cases of autism spectrum disorder (ASD) in the hemizygous state in males (Pinto et al., 2010; Piton et al., 2011; Iossifov et al., 2014; Pilorge et al., 2016). To date, a microdeletion (*GLRA2*∆ex8-9) and two *de novo* missense mutations p.N109S and p.R126Q (p.N136S and p.R153Q in the GlyR α2 subunit with signal peptide) have been functionally characterized (Pilorge et al., 2016). *GLRA2*∆ex8-9 resulted in the production of a truncated mRNA that escaped nonsense-mediated RNA decay. However, this resulted in a truncated GlyR α2 subunit protein lacking the third and fourth membrane-spanning domains and the cytoplasmic M3–M4 intracellular loop. Functional studies revealed that GlyR α2^∆ex8-9^ was not expressed at the cell surface in CHO cells (Pilorge et al., 2016). By contrast, the GlyR α2^N109S^ and α2^R126Q^ mutations resulted in reduced cell-surface expression and substantially reduced glycine sensitivity by one-to-two orders of magnitude (Pilorge et al., 2016). These mutations were classified as loss-of-function based on their impaired trafficking and inability to respond to physiological levels of glycine. The aim of this study was to functionally characterize a third missense variant in *GLRA2*, (p.R323L), where the pathomechanism was unclear since it was found in the heterozygous state in a female patient and inherited from an apparently healthy mother (Piton et al., 2011).

## Materials and Methods

### Cell Culture and Molecular Biology

Expression constructs encoding the human GlyR α2 and β subunits were combined in 1α:50β ratio (heterosynapse and macropatch recordings) or 1α:100β (single-channel recordings) and transfected into HEK293 cells via Ca^2+^ phosphate-DNA co-precipitation. This resulted in a high level of expression of heteromeric α2β GlyRs (Zhang Y. et al., 2015). For heterosynapse experiments, expression constructs for the mouse neuroligin 2A (NL2A) splice variant and rat gephyrin were co-transfected along with GlyR constructs to facilitate the formation of heterosynapses. Empty pEGFP or CD4 plasmid was also transfected as expression markers. Site-directed mutagenesis was performed using the QuikChange kit (Agilent), and the successful incorporation of mutations was confirmed by Sanger DNA sequencing. Mutation position in the GlyR α2 subunit is indicated using mature subunit numbering (i.e., after signal peptide cleavage).

### Heterosynapse Formation

Primary cultures of spinal cord neurons were prepared as previously described (Dixon et al., 2015; Zhang Y. et al., 2015). E15 timed-pregnant rats were euthanized via CO_2_ inhalation in accordance with procedures approved by the University of Queensland Animal Ethics Committee. Cells were plated at a density of ~80,000 cells per 18 mm poly-D-lysine coated coverslip in DMEM medium with 10% (v/v) foetal bovine serum. After 24 h, the plating medium was changed to Neurobasal medium supplemented with 2% (v/v) B27 and 1% (v/v) GlutaMAX, and a second feed after 1 week replaced half of this medium. Neurons were grown for 1–4 weeks *in vitro* and heterosynaptic co-cultures were prepared by directly introducing transfected HEK293 cells onto the primary neuronal cultures 1–3 days prior to recording.

### Electrophysiology

Whole-cell recordings were performed on transfected HEK293 cells in voltage-clamp mode using a HEKA EPC10 amplifier (HEKA Electronics, Lambrecht, Germany) and PATCHMASTER software (HEKA), at room temperature. Cells were placed in an external solution comprising (in mM): 140 NaCl, 5 KCl, 2 CaCl_2_, 1 MgCl_2_, 10 HEPES and 10 D-glucose, adjusted to pH 7.4 with NaOH. Patch pipettes (1–3 MΩ resistance), were pulled from borosilicate glass (GC150F-7.5, Harvard apparatus), and filled with an intracellular solution containing the following (in mM): 145 CsCl, 2 CaCl_2_, 2 MgCl_2_, 10 HEPES, 10 EGTA and 2 MgATP, adjusted to pH 7.4 with NaOH. Glycine-gated currents were recorded at a holding potential of −40 mV, digitized at 4 kHz and filtered at 10 kHz. For inhibitory postsynaptic current (IPSC) recordings, patch-pipette resistances were adjusted to 4–6 MΩ and filled with the same internal solution. Series resistance was routinely compensated to 60% of maximum and was monitored throughout the recording. Both spontaneous and action potential-evoked glycinergic IPSCs in HEK293 cells were recorded at a holding potential −60 mV and signals were digitally sampled at 10 kHz and filtered at 4 kHz. As these IPSCs were completely abolished by 1 μM tetrodotoxin (not shown), we infer they were induced by spontaneous action potentials.

Single-channel currents were recorded from outside-out excised patches at a clamped potential of −70 mV. Glass electrodes were pulled from borosilicate glass (G150F-3; Warner Instruments), coated with a silicone elastomer (Sylgard-184; Dow Corning) and heat-polished to a final tip resistance of 8–15 MΩ when filled with an intracellular solution containing (in mM) 145 CsCl, 2 MgCl_2_, 2 CaCl_2_, 10 HEPES and 5 EGTA, pH 7.4. Excised patches were directly perfused with extracellular solution by placing them in front of one barrel of a double-barrelled glass tube. Single-channel currents were either recorded while the patch was exposed to extracellular solution (without added glycine) or elicited by exposing the patch continuously to glycine (100 μM or 3 mM) containing solution. Experiments were recorded using an Axopatch 200B amplifier (Molecular Devices), filtered at 5 kHz and digitized at 20 kHz using Clampex (pClamp 10, Molecular Devices) via a Digidata 1440A digitizer. The currents were filtered off-line at 3 kHz for analysis.

Macropatch recordings were performed in the excised outside-out patch-clamp configuration. Patch pipettes were fire-polished to a resistance of approximately 10 MΩ and filled with the same internal solution. Macroscopic currents in outside-out patches pulled from transfected HEK293 cells were activated by brief (<1 ms) exposure to agonists using a piezo-electric translator (Siskiyou). The speed of the solution exchange system was regularly calibrated by rapidly switching the solution perfusing an open patch pipette between standard extracellular solution and an extracellular solution that had been diluted by 50% with distilled water. By monitoring the resulting pipette current, we were able to ensure that the solution perfusing the macropatch was completely exchanged within 200 μs (Dixon et al., 2014). Recordings were performed using a Multiclamp 700B amplifier and pClamp9 software (Molecular Devices), filtered at 4 kHz and sampled at 10 kHz.

### Analysis

Analyses of IPSC amplitudes, 10%–90% rise times, and weighted decay time constants were performed using Axograph (Axograph Scientific). Only cells with a stable series resistance of <25 MΩ throughout the recording period were included in the analysis. Single peak IPSCs with amplitudes of at least three times above the background noise were detected using a semi-automated sliding template. Each detected event was visually inspected and only well-separated IPSCs with no inflections in the rising or decay phases were included. To calculate macroscopic current decay time constants, averaged macroscopic traces were fitted with double-exponential functions in Axograph X, and a weighted time constant was calculated from individual time constants (τ1, τ2) and their relative amplitude (A1, A2) as follows: *τ*_weighted_ = (τ1×A1 + τ2×A2)/(A1 + A2). The averaged data from individual cells were then pooled to obtain group data. Statistical analysis, and plotting were performed with Prism 5 (GraphPad Software). The fitting of single Gaussian functions to IPSC amplitude and decay time constant distributions was also performed using Prism 5. All data are presented as mean ± SEM. Student’s unpaired *t-tests* or one-way ANOVAs, as appropriate, were employed for comparisons. For all tests, the number of asterisks corresponds to level of significance: *p < 0.05, **p < 0.01, ***p < 0.001 and ****p < 0.0001. Single-channel recordings were analyzed with pClamp 10 (Clampfit, Molecular Devices) or QuB software. Segments of single-channel activity separated by long periods of baseline were idealized into noise-free open and shut events using a temporal resolution of 70 μs. Single-channel activations were separated using a shut period (*t*_crit_) ranging between 6 ms and 30 ms, however most of the *t*_crit_ values were <10 ms. Group data form current-voltage experiments were fitted to a polynomial using Sigmaplot (Systat Software), from which reversal potential was obtained. Ohm’s Law was used to find single-channel conductance (γ), in which *V*_hold_ is the holding potential (−70 mV), *V*_ljp_ is the liquid junction potential (4.7 mV for the solutions used) and *V*_rev_ is the reversal potential as follows: γ = (*i*)/(*V*_hold_ − *V*_ljp_ − *V*_rev_).

## Results

### GlyR α2^R323L^ Mutation Is Associated with Autism, Loss of Acquired Language, Seizures, Macrocephaly and Hypothyroidism

**Figure 1:**
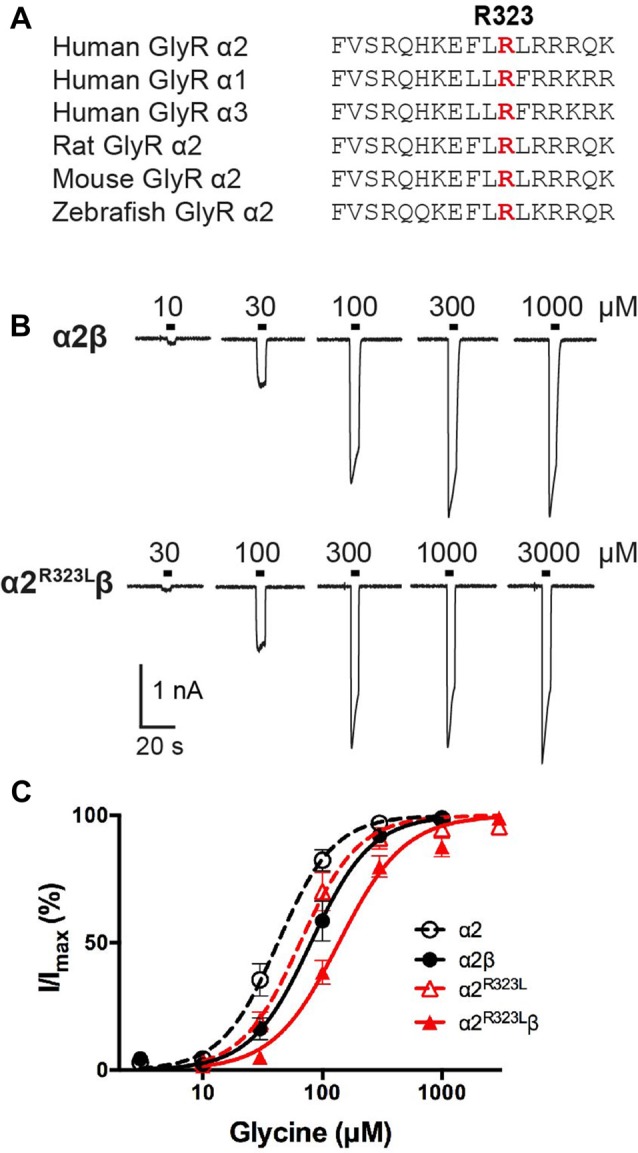
**Effect of the Glycine receptor (GlyR) α2 subunit p.R323L mutation examined by whole-cell patch-clamp recording. (A)** Alignments of vertebrate GlyR α2 subunit amino acid sequences indicating the conserved arginine residue at position 323 (red). **(B)** Sample whole-cell recordings for heteromeric α2β and α2^R323L^β GlyRs in the presence of indicated glycine concentrations. **(C)** Averaged whole-cell glycine dose-response curves for α2, α2β, α2^R323L^ and α2^R323L^β GlyRs.

Previously reported *GLRA2* mutations associated with ASD include a microdeletion (*GLRA2*∆ex8-9; Pinto et al., 2010) and *de novo* missense mutations p.N109S (Iossifov et al., 2014) and p.R126Q (Pilorge et al., 2016) identified in the hemizygous state in males with non-syndromic autism. Additional clinical symptoms were noted in two of these individuals, including language delay with functional language and low average IQ (*GLRA2*∆ex8-9) and hyperactivity, severe language delay and tonic-clonic seizures (p.R126Q). These mutations were classified as loss of function based on impaired cell-surface expression of GlyR α2, and abolition (*GLRA2*∆ex8-9) or markedly reduced sensitivity to glycine (p.N109S and p.R126Q; Pilorge et al., 2016). These recessive mutations were not found to be disease causing in female carriers, due to the presence of a normal *GLRA2* allele, and because *GLRA2* escapes X-inactivation in the vast majority of tissues including brain (Cotton et al., 2015). However, many mutations in the GlyR α1 subunit gene (*GLRA1*) that cause startle disease show dominant inheritance (Harvey et al., 2008; Bode and Lynch, 2014). We therefore decided to examine the functional effects of potentially pathogenic variants found in females. In particular, we focussed on p.R323L (p.R350L in the GlyR α2 subunit with signal peptide; c.1049G>T in NM_001118885). This missense mutation was previously reported in a female with ASD (Piton et al., 2011) that was predicted to be damaging by a number of software packages (including PolyPhen-2, SNPs&GO, MutPred, PANTHER and SIFT; Pilorge et al., 2016). This mutation is also extremely rare, as it is not reported in the Genome Aggregation Database (gnomAD^1^) currently comprising 126,216 exome sequences and 15,136 whole-genome sequences from unrelated individuals. The mutated arginine is located the large M3–M4 intracellular loop of the GlyR α2 subunit and is highly conserved among GlyR subunits (Figure 1A). Additional clinical symptoms reported in this case include loss of acquired words, seizures, mild motor developmental delay, macrocephaly and hypothyroidism (Gauthier, personal communication).

### Effects of the α2^R323L^ Mutation on GlyR Channel Properties

To determine the functional effects of the GlyR α2^R323L^ mutation, we examined the potency of glycine in activating recombinant homomeric α2^R323L^ and heteromeric α2^R323L^β receptors. Figure 1B illustrates whole-cell currents recorded in response to increasing concentrations of glycine in HEK293 cells expressing wild-type GlyRs or those containing α2^R323L^. The glycine dose-response curve of both homomeric α2^R323L^ and heteromeric α2^R323L^β GlyRs were modestly right-shifted, with EC_50_ values of 67.9 ± 13.4 (α2^R323L^) and 143.0 ± 12.9 μM (α2^R323L^β), compared with 46.6 ± 6.7 and 90.3 ± 14.6 μM for the corresponding wild-type α2 and α2β GlyRs (Table 1, Figure 1C). Thus, in both homomeric and heteromeric GlyRs, the p.R323L mutation results in a small decrease in apparent glycine sensitivity. There was no significant difference in peak whole-cell currents in all the receptors tested (range, ~2–1.5 nA, Table 1) suggesting that mutations of R323 do not alter GlyR cell-surface expression.

**Table 1:** **Summary of dose-response analysis data for α2, α2β, α2^R323L^ and α2^R323L^β Glycine receptors (GlyRs)**.

	α2	α2β	α2^R323L^	α2^R323L^β
EC_50_ μM	46.6 ± 6.7	90.3 ± 14.6	67.9 ± 13.4	143.0 ± 12.9*
Hill slope	2.2 ± 0.1	2.1 ± 0.1	2.1 ± 0.2	2.0 ± 0.3
I_max_ (nA)	1.6 ± 0.2	2.1 ± 0.3	1.5 ± 0.4	1.7 ± 0.2
*n*	12	11	9	13

Statistical comparisons of α2^R323L^ and α2^R323L^β were made relative to α2 and α2β GlyRs, respectively, via an unpaired Student’s *t*-test. *p < 0.05.

### The GlyR α2^R323L^ Mutation Alters Intrinsic Channel Gating

**Figure 2:**
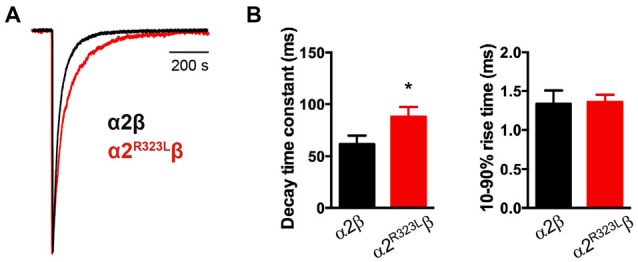
**Glycine-evoked α2^R323L^β currents have slower intrinsic kinetic properties compared to wild-type α2β. (A)** Averaged macropatch currents recorded from outside-out patches containing α2β (black trace) and α2^R323L^β (red trace) GlyRs. Currents were activated by brief (~1 ms) exposure to saturating (3 mM) glycine. To allow comparison of kinetic properties, the currents were normalized to the same peak amplitude. **(B)** Comparison of mean macropatch current decay time constants and 10%–90% rise times. *p < 0.05 relative to α2β GlyRs via unpaired Student’s *t*-test.

This modest change in dose-response relationship is insufficient to explain the pathogenic effects of the p.R323L mutation, especially since the peak glycine concentration in the synaptic cleft is thought to reach 1–3 mM in embryonic zebrafish neurons (Legendre, 1998) and 2.2–3.5 mM in adult rat spinal neurons (Beato, 2008). Clearance of glycine away from the cleft has also been estimated to occur on a 0.6–0.9 ms time scale (Beato, 2008). Based on these parameters, we examined intrinsic channel properties by rapidly applying saturating glycine (3 mM) for a period of ~1 ms to excised outside-out HEK293 cell patches expressing wild-type and mutant α2^R323L^β receptors. Sample macroscopic currents are shown in Figure 2A. The time course of deactivation was fitted by a double exponential function with a mean time constant of 61.9 ± 3.2 ms (*n* = 15) for α2β GlyRs and a mean time constant of 87.9 ± 5.4 ms (*n* = 17) for α2^R323L^β GlyRs (Figure 2B, Table 2). The deactivation time constant for α2^R323L^β receptors was ~1.5-fold slower compared to wild-type GlyRs (*p* < 0.05). However, the rise time did not differ between wild-type and mutant receptors (α2β, 1.3 ± 0.1 ms; α2^R323L^β, 1.4 ± 0.2 ms; *p* > 0.05). This suggests that the p.R323L mutation in M3–M4 loop enhances channel function by slowing the channel closing rate. An analysis of the individual components of the double exponential fit revealed that the longer time constant and the fraction of the total current it represents increased (Table 3).

**Table 2:** **Comparison of 10%–90% rise times, decay time constants and maximal peak currents of inhibitory postsynaptic currents (IPSCs) and macropatch currents mediated by the wild type and mutant GlyRs**.

		α2β	α2^R323L^β	α2^R323A^β	α2^R323K^β	α2^R323I^β
10%–90% rise time (ms)	IPSCs	2.6 ± 0.3 (7)	4.9 ± 0.8** (6)	2.5 ± 0.3 (4)	2.3 ± 0.3 (7)	2.4 ± 0.1 (5)
	macropatch currents	1.4 ± 0.1 (17)	1.3 ± 0.2 (15)	–	–	–
Deactivation time constant (ms)	IPSCs	27.0 ± 1.5	60.8 ± 4.9****	37.1 ± 6.8	32.3 ± 2.8	32.0 ± 2.5
	macropatch currents	61.9 ± 3.2	87.9 ± 5.4*	–	–	–
*I*_max_ (pA)	IPSCs	32.8 ± 2.5	34.0 ± 11.9	24.3 ± 4.7	28.5 ± 3.3	18.2 ± 2.0
	macropatch currents	381 ± 47	330 ± 76	–	–	–

*p < 0.05, **p < 0.01 and ****p < 0.0001 relative to the wild-type α2β GlyR, unpaired t-test for comparisons between two groups and one-way ANOVA followed by Bonferroni’s post hoc correction for multiple comparisons. For IPSCs, n values refer to the total number of cells from which data were collected. For each cell, parameters were analyzed from a single IPSC waveform that was digitally averaged from >50 individual events.

**Table 3:** **Individual time constants and relative areas for macropatch currents**.

Receptor	τ1 (ms)	A1	τ2 (ms)	A2	*n*
Wild-type α2 GlyR	132 ± 7*	0.29 ± 0.03	34.4 ± 2.6	0.71 ± 0.03	8
α2^R323L^β GlyR	173 ± 14*	0.37 ± 0.03	37.7 ± 2.8	0.63 ± 0.02	7

Data represent mean ± SEM. *p < 0.05. The relative areas are expressed as a fractions.

### The GlyR α2^R323L^ Mutation Alters Glycinergic IPSC Kinetics

**Figure 3:**
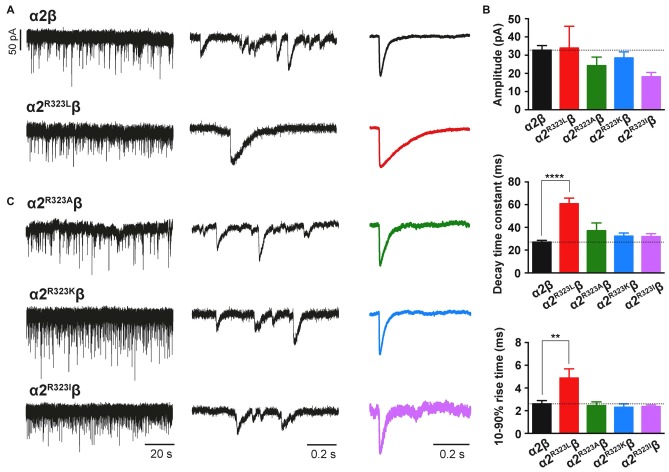
**The GlyR α2^R323L^ mutation results in prolonged inhibitory postsynaptic currents (IPSCs) in inhibitory heterosynapses. (A)** Representative recordings of glycinergic IPSCs in HEK293 cells expressing α2β and α2^R323L^β GlyRs at different temporal resolutions. Normalized IPSCs each averaged from >50 events from the corresponding cell are shown in the right panel (color traces).** (B)** Comparison of mean IPSC amplitude, decay time constant and 10%–90% rise time for the indicated GlyRs. **(C)** Representative recordings of glycinergic IPSCs in HEK293 cells expressing the α2^R323A^β, α2^R323K^β and α2^R323I^β GlyRs at different temporal resolutions. Recordings were performed at −60 mV. Statistical significance was determined via one-way ANOVA followed by Bonferroni’s *post hoc* correction with significance represented by **p < 0.01 and ****p < 0.0001 relative to α2β GlyRs.

To test whether the enhancement of GlyR function altered glycinergic transmission, we used a heterosynapse system that allows control over the subunit composition of GlyRs in glycinergic synapses (Zhang Y. et al., 2015). We inserted α2β and α2^R323L^β GlyRs into heterosynapses in turn to evaluate the properties of the resulting IPSCs. Sample IPSC recordings from heterosynapses incorporating α2β and α2^R323L^β isoforms are shown at different temporal resolution in Figure 3A with averaged normalized IPSCs. The averaged IPSC amplitudes, 10%–90% rise times and decay time constants for α2β and α2^R323L^β GlyRs are presented in Figure 3B and Table 2. We observed that IPSCs mediated by α2^R323L^β receptors displayed a 2-fold slower rise and decay time than those mediated by wild-type α2β GlyRs (10%–90% rise time: α2β, 2.6 ± 0.3 ms, *n* = 7; α2^R323L^β, 4.9 ± 0.8 ms, *n* = 6, *p* < 0.01; decay time: α2β, 27.0 ± 1.5 ms; α2^R323L^β, 60.8 ± 4.9 ms, *p* < 0.0001), whereas the mean IPSC amplitude did not differ significantly (α2β, 32.8 ± 2.5 pA; α2^R323L^β, 34.0 ± 11.9 pA; *p* > 0.05). We also sought to investigate the effects of the previously described autism mutations, α2^N109S^ and α2^R126Q^ (Pilorge et al., 2016), in heterosynapses, but heteromeric GlyRs incorporating these mutations yielded no detectable synaptic currents (*n* > 10 cells expressing each mutant GlyR). This result was expected given that both mutations dramatically increased the glycine EC_50_ (Pilorge et al., 2016).

### The Nature of the Substitution at R323 Governs the Time-Course of Glycinergic IPSCs

We next sought to investigate whether the nature of the side chain, charge or the steric characteristics of the p.R323L substitution contributes directly to the observed increase in intrinsic channel deactivation rates. Arginine is basic and polar (positively charged) with a 3-carbon aliphatic straight chain, capped at the distal end by a complex guanidinium group. We replaced R323 by alanine (α2^R323A^) which is aliphatic, uncharged and has a short side chain consisting of a single methyl group. We also examined substitutions with lysine (α2^R323K^, which is basic, and has a positively charged ε-amino group) and isoleucine (α2^R323I^, aliphatic, non-polar and differing from leucine only in the position of a side chain methyl group). We then characterized the effects of each GlyR α2 mutant on the kinetics of heterosynaptic IPSCs. Figure 3C shows sample recordings from heterosynapses incorporating the GlyR α2^R323A^, α2^R323K^ and α2^R323I^ constructs, and averaged normalized IPSCs are presented in the right panel. All three substitutions produced heterosynaptic IPSCs that had kinetics similar to those of wild-type α2β GlyRs. Again, similar peak currents between mutant and wild-type receptors in macropatch and heterosynapse recordings suggests comparable surface expression (Table 2).

### The GlyR α2^R323L^ Mutation Alters GlyR Single-Channel Kinetics

**Figure 4:**
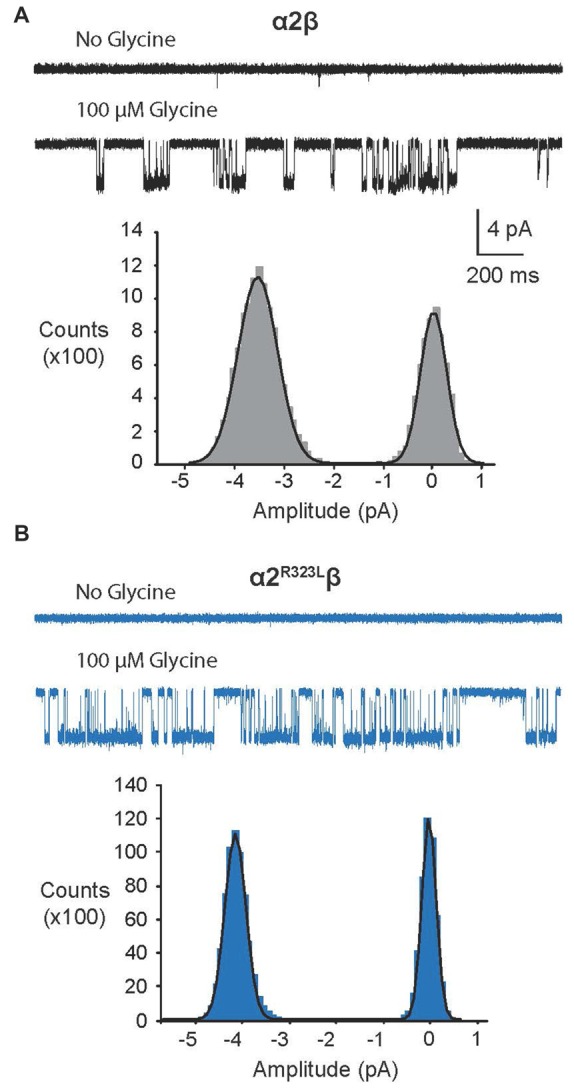
**Single-channel amplitude of heteromeric α2β and α2^R323L^β GlyRs. (A,B)** Both α2β and α2^R323L^β GlyRs showed no significant spontaneous activity in glycine-free solution (above). Activity in α2β and α2^R323L^β receptors was observed in 100 μM glycine solution (below). Amplitude histogram of α2β and α2^R323L^β (bottom panels) revealed that the mutation produced a small but consistent increase in single-channel amplitude (α2β, ~3.2 pA, α2^R323L^β ~3.8 pA). Recordings were performed at −70 mV and channel openings are shown as downward deflections.

**Figure 5:**
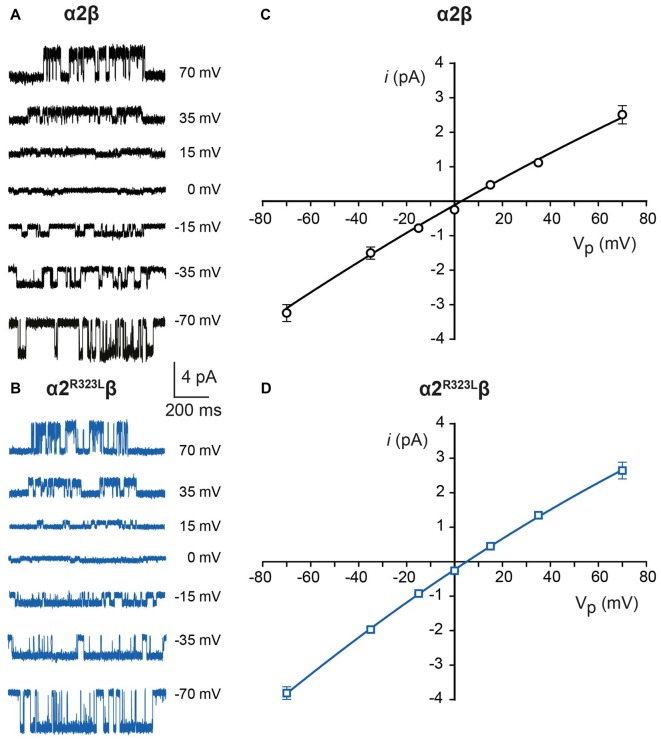
**Current-voltage and conductance characteristics of heteromeric α2β and α2^R323L^β GlyRs**. Sample currents obtained at the indicated pipette voltages for heteromeric α2β **(A)** and α2^R323L^β GlyRs **(B)**. Current-voltage relationships of heteromeric α2β **(C)** and α2^R323L^β** (D)** GlyRs, obtained from averaged data from three to five patches.

Since another pathomechanism associated with GlyR mutations is spontaneously-opening channels (Chung et al., 2010; Bode et al., 2013; James et al., 2013; Zhang et al., 2016), we examined the activity of α2β and α2^R323L^β GlyRs in outside-out patches. No spontaneous activity was observed when patches containing α2β and α2^R323L^β GlyRs were perfused with glycine-free solution for more than 1 min (Figures 4A,B above). However, at a saturating 100 μM glycine concentration, active periods were induced and observed as clusters of openings (Figures 4A,B). The current amplitude at −70 mV was determined for α2β and α2^R323L^β GlyRs by plotting amplitude histograms and fitting these to Gaussian functions. GlyRs containing the α2β subunit combination displayed currents of ~3.2 pA, whereas single-channel currents for α2^R323L^β GlyRs were ~3.8 pA at −70 mV (Figures 4A,B below). The single-channel conductance was determined for both α2β and α2^R323L^β GlyRs by carrying out current-voltage experiments over a range of voltages from −70 mV to +70 mV and averaged from three to five patches (Figures 5A,B). Mild inward rectification was observed in the current-voltage plots of both channels (Figures 5C,D). Current-voltage plots for heteromeric α2β and α2^R323L^β GlyRs intersected the voltage axis at ~+3 mV (Figure 5C) and ~+5.5 mV (Figure 5D), respectively. Mean single-channel conductance levels were calculated to be 41.2 pS for α2β and 47.5 pS for α2^R323L^β and were statistically different (*p* < 0.05). Thus, the conductance of α2^R323L^β GlyRs is slightly larger than that for wild-type α2β GlyRs, but less than that previously reported for homomeric α2 subunit GlyRs (60–120 pS; Wang et al., 2006; Krashia et al., 2011).

**Figure 6:**
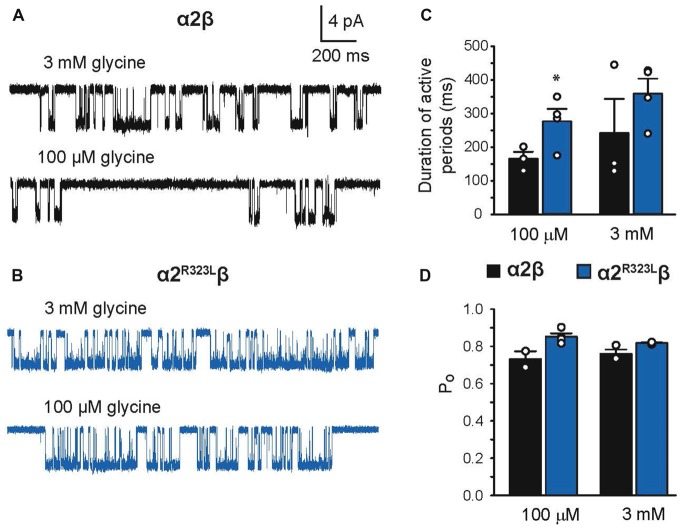
**Duration of single-channel activations in heteromeric α2β and α2^R323L^β GlyRs**. Single-channel currents at the indicated concentrations of glycine for patches expressing heteromeric α2β **(A)** and α2^R323L^β GlyRs **(B)**. Bar plots of the durations of active periods **(C)** and open probability **(D)** at the indicated glycine concentrations. Note that mutant α2^R323L^β GlyRs activate in clusters with a longer opening duration compared to wild-type α2β GlyRs. *p < 0.05.

Finally, we investigated single-channel kinetics for α2β and α2^R323L^β GlyRs. Samples of opening durations for α2β and α2^R323L^β are shown in Figures 6A,B, respectively. The duration of activations and open probabilities (*P*_o_) for two channel types were assessed at 100 µM and 3 mM glycine (Table 4). At both concentrations, the mean duration of active periods for α2^R323L^β GlyRs was longer than that observed for α2β GlyRs (Figure 6C). It is noteworthy that there was a high standard deviation in the values for wild-type α2β GlyRs at 3 mM glycine. Although the *P*_o_ values of the two receptors were indistinguishable at 3 mM glycine, at 100 µM glycine α2^R323L^β showed a significant increase in *P*_o_ compared to α2β (Figure 6D).

**Table 4:** **Single-channel activation parameters of α2β and α2^R323L^β receptors**.

	Opening probabilities (*P*_o_)		Mean duration of active periods (ms)	
Channel	100 μM glycine	3 mM glycine	100 μM glycine	3 mM glycine
α2β	0.73^#^	0.76 ± 0.02	165 ± 21	242 ± 102
α2^R323L^β	0.85 ± 0.02	0.82 ± 0.02	277 ± 37*	360 ± 44

Data represent mean (*n* = 3–4) ± SEM. *p < 0.05, ^#^from *n* = 2 patches.

In summary, the increase in decay times for synaptic and macropatch currents, the small but significant increase in single-channel conductance and the prolongation of individual receptor active periods clearly demonstrates that overall, the R323L mutation confers a gain-of-function.

## Discussion

We have described the detailed functional characterization of the GlyR α2 subunit p.R323L mutation associated with autism, macrocephaly, loss of acquired language, epilepsy and hypothyroidism. We used a combination of rapid glycine application, heterosynapses and single-channel recordings to quantify the intrinsic channel properties and IPSC kinetic properties of α2^R323L^β GlyRs. We found that the p.R323L mutation resulted in a small apparent decrease in glycine sensitivity in α2^R323L^ and α2^R323L^β GlyRs (Figure 1, Table 1). Notably, this was not of the same order of magnitude previously observed for the GlyR α2^N109S^ and α2^R126Q^ loss-of-function mutations (Pilorge et al., 2016). Using outside-out patches, we found that the p.R323L mutation did not alter rise time, but slowed the deactivation time constant of α2^R323L^β GlyRs by ~1.5-fold compared to wild-type α2β GlyRs (Figure 2, Table 2). We also examined properties of GlyR α2^R323L^β GlyRs in glycinergic heterosynapses that were formed between presynaptic terminals of cultured spinal glycinergic interneurons and HEK293 cells expressing recombinant GlyRs. As well as allowing control over the subunit composition of the GlyRs under study, the electrotonically compact shape of HEK293 cells allows IPSC waveforms to be resolved with high fidelity (Dixon et al., 2015; Zhang Y. et al., 2015). IPSCs mediated by α2^R323L^β receptors in heterosynapses displayed a 2-fold slower rise and decay time than those mediated by wild-type α2β GlyRs, whereas the mean IPSC amplitude did not differ significantly (Figure 3, Table 3). As the change in IPSC decay rates corresponds well with the duration of single receptor active periods in the α2^R323L^β receptors, we infer that the slower decay of IPSCs is dominated by the intrinsic gating properties of the channels. We next sought to understand whether the nature of the amino acid at position 323 is vital for the effect on ion channel function, replacing the basic and positively charged R323 by alanine (aliphatic, uncharged, short side chain), lysine (also basic and positively charged) and isoleucine (aliphatic, non-polar) and measuring the effects on heterosynaptic IPSCs. Surprisingly, all three substitutions resulted in GlyRs with wild-type characteristics (Figure 3, Table 2). This suggests that the bulky side chain at position R323 did not generate steric interactions with the adjacent GlyR subunits, and electrostatic mechanisms are not responsible for prolonged IPSC time courses observed in α2^R323L^β GlyRs.

Finally, we examined the properties of α2β and α2^R323L^β GlyRs using single-channel recordings (Figures 4–6), excluding the possibility that these mutant GlyRs resulted in spontaneously-opening channels, a known pathomechanism in startle disease involving GlyR α1β dysfunction (Chung et al., 2010; Bode et al., 2013; James et al., 2013; Zhang et al., 2016). Mean single-channel conductances were 41.2 pS for α2β and 47.5 pS for α2^R323L^β (Table 3) suggesting that the conductance of α2^R323L^β GlyRs is slightly, but significantly larger than that for wild-type α2β GlyRs. Moreover, the duration of activations and opening probability (*P*_o_) at 100 μM were also increased (Figure 6, Table 3), suggesting that α2^R323L^β GlyRs spend more time in conducting states while active and were active for longer periods than wild-type α2β GlyRs. A single-channel study that examined α2 homomeric GlyRs concluded that these receptors exhibited longer active periods (mean open times) compared to adult synaptic α1β heteromeric GlyRs (Krashia et al., 2011). Another study that measured the duration of single-channel active periods estimated a mean duration of ~500 ms for α1β heteromeric GlyRs, however, the active periods were isolated using longer shut periods (*t*_crit_) (Scott et al., 2015). Taken together, these data strongly suggest that the p.R323L mutation results in an overall *gain-of-function*, although uniquely the resulting GlyRs do not show spontaneous channel openings. This may explain why this mutation is pathogenic even in the heterozygous state, and relates to some of the unique clinical features seen in this individual, including autism, macrocephaly and epilepsy. Autism has been associated with signs of cortical enlargement in children as young as 6 months of age and precedes brain overgrowth observed at 1–2 years of age (Hazlett et al., 2017). Autism, language delay and seizures have previously been associated with other human GlyR α2 mutations (Pilorge et al., 2016) and mouse models suggest that these features could arise from defects in cortical neuronal migration and/or dendritic branching, resulting in disrupted excitatory/inhibitory balance (Avila et al., 2013, 2014; Morelli et al., 2017). Given that loss-of-function of GlyR α2 leads to microcephaly in knockout mice (Avila et al., 2014), we speculate that the overall gain-of-function we observed for GlyR α2^R323L^ results in macrocephaly in the index patient.

One question that remains is why the p.R323L mutation is pathogenic in the index patient, but not in her mother, who is also a heterozygous carrier of the mutation. Here we note that although GlyR dysfunction has not previously been linked to hypothyroidism, this condition delays the development of the hyperpolarizing shift in the Cl^−^ equilibrium potential during neuronal development, which in turn delays the maturation of GABAergic and glycinergic synaptic inhibition (Friauf et al., 2008). This is thought to explain the link between thyroid hormone deficiency and functional deficits in the nervous system (Friauf et al., 2008). Thus, one possibility is that the hypothyroidism in this patient could delaying the switch from excitatory to inhibitory synaptic transmission, thus exacerbating the effects of the GlyR α2 p.R323L mutation and prolonging the period of disrupted cortical neuronal migration. This hypothesis could be tested in future knock-in models for GlyR α2^R323L^ by measuring cortical neuronal migration and progenitor homeostasis, since hypothyroidism can be induced in animal models (Friauf et al., 2008).

**Figure 7:**
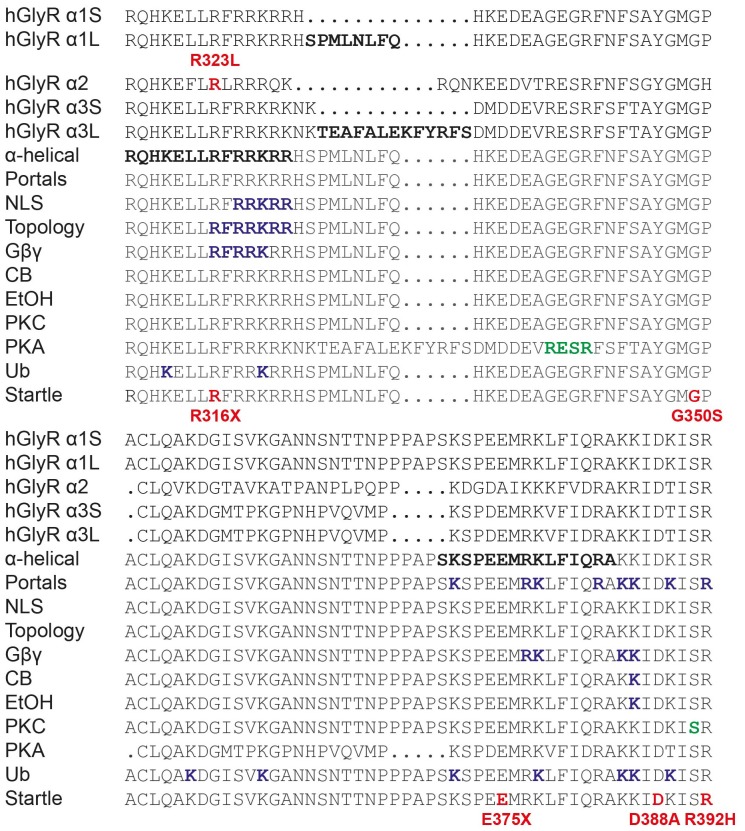
**Functionally-important residues and disease-causing mutations in GlyR intracellular loops**. M3–M4 loop sequences of human GlyR α1-α3 subunits and alternatively spliced variants (α1S, α1L, α3S and α3L) are shown. Bold letters indicate residues that have been investigated *in vitro* for: structure (α-helical elements), ion permeation and desensitization (portals), nuclear localization signals (NLS), GlyR topology, binding of intracellular proteins (Gβγ) or pharmacological agents such as cannabinoids (CB) and ethanol (EtOH), phosphorylation by PKC or PKA or ubiquitination (Ub). Mutations found in human patients with autism (GlyR α2) or startle disease (GlyR α1) are indicated in bold red type. Modified from Langlhofer and Villmann (2016).

Our study also underlines the importance of the GlyR intracellular M3–M4 loop in GlyR function (Langlhofer and Villmann, 2016). This region in GlyRs has been studied extensively (Figure 7). It is known to have α-helical elements (Burgos et al., 2015) and is involved in phosphorylation (Harvey et al., 2004; Manzke et al., 2010; Han et al., 2013), intracellular sorting (Melzer et al., 2010), protein-protein interactions (Meyer et al., 1995; Melzer et al., 2010; Del Pino et al., 2014; Burgos et al., 2015), subunit topology (Sadtler et al., 2003) and modulation by Gβγ, ethanol and cannabinoids (CB; Yevenes et al., 2008; Yevenes and Zeilhofer, 2011; Burgos et al., 2015; Sanchez et al., 2015). Others have highlighted the importance of the GlyR M3–M4 intracellular loop in desensitization behavior (Nikolic et al., 1998), channel gating (Breitinger et al., 2009) and conductance (Carland et al., 2009). Of these, the GlyR α2 p.R323L substitution only overlaps with motifs involved in GlyR topology and/or Gβγ modulation in the GlyR α1 subunit. Since our data demonstrate that the GlyR α2^R323L^ reaches the membrane and is functional, it is unlikely that this mutation drastically affects GlyR topology. In addition, GlyR α2 is not modulated by Gβγ (Yevenes et al., 2010). Lastly, GlyR α2 p.R323L does not correspond to the position of any known startle disease missense mutation located in the M3–M4 loop (Figure 7), although a nonsense mutation (p.R316X) was reported (Tsai et al., 2004) at the same position in the GlyR α1 subunit. Given the effects of the GlyR α2 p.R323L mutation on channel conductance properties, intrinsic channel gating and IPSC kinetics, we suggest that this residue is a potential determinant of theoretical intracellular portals consisting of charged residues influencing ion permeation and conductance (Carland et al., 2009). Previously, these elements were thought to be localized at the C-terminal end of the GlyR α1 subunit M3–M4 loop, where mutation of selected positively-charged residues (e.g., R377, K378, K385 and K386) to negatively-charged residues gave rise to non-functional channels (Carland et al., 2009). Our study provides new evidence suggesting that positively-charged residues at the N-terminal end of the M3–M4 loop may have a key role to play in the control of IPSC rise and decay times, and mean channel opening durations.

## Author Contributions

JWL, RJH and AK designed the experiments; YZ, TNTH and AK performed the experiments; JWL, RJH and AK analyzed the data and wrote the article. All authors were involved in revising the article for important intellectual content, and gave final approval of the version to be published.

## Funding

This work was supported by the National Health and Medical Research Council of Australia (1058542 to JWL), the Australian Research Council (DP150102428 to JWL) and the Medical Research Council (J004049, M013502 to RJH). The funders had no role in study design, data collection and analysis, decision to publish, or preparation of the manuscript.

## Conflict of Interest Statement

The authors declare that the research was conducted in the absence of any commercial or financial relationships that could be construed as a potential conflict of interest.

## Footnotes

^1^http://gnomad.broadinstitute.org/
